# The Treatment of Gonorrhœa

**Published:** 1892-08

**Authors:** John A. Wyeth

**Affiliations:** At the N. Y. Polyclinic


### THE TREATMENT OF GONORRHŒA.

BY DR. JOHN A. WYETH, AT THE N. Y. POLYCLINIC.

My idea about gonorrhœa is that we should treat it just as we do an acute abcess; that is, by drainage, with just as much asepsis as possible. Let the urethra hang downwards, with a bag of some sort loosely attached to it to catch the discharge. Do not have it tightly bandaged or plugged up with cotton. In a specific urethritis, where the inflammation has extended into the deeper layers of the epithelium, the various bich oride and zinc injections are not likely to do much good. In an ordinary, non-specific urethritis, however, where the inflammation is superficial, you can inject with good results. There are certain remedies which will increase the amount of urine and render it aseptic, so that you can use the bladder as an irrigator in gonorrhœal inflammation. Among these remedies is boracic acid, or, better still, the oil of gaultheria This drug will absolutely sterilize the urine. You can give five or six drops every three or four hours. During the later stages of the inflammation, mild solutions of the sub-acetate of lead can be used, which will help keep the urethra clean. A syringe with a short nozzle should be employed, so as to only just enter the meatus. The injection should be of moderate size, so as not to over-distend the urethra. During the acute stages, I instruct my patient to take a warm sitzbath night and morning. When the acute stage has subsided, the administration of some of the so-called blennorhetics will be found beneficial.

I have found the capsules containing santal-midy, a preparation made from sandal-wood, a very good thing. I consider these superior to the old-time cubeb preparations, which are liable to disturb digestion.—*Ex.*

---

ASAFŒTIDA is recommended in habitual abortion not due to syphilis or disease of the uterus and its appendages, by Dr. Guido Turazza, in *Centralblatt fur Gynœcologie.* He administers it in 1¼ gr pills every other day at first, and increases the interval gradually to ten days during the whole period of gestation.—*Notes on New Remedies.*

THE TREATMENT OF GONORRHŒA.—My treatment of gonorrhœa in all stages has for long been very monotonous. Almost without regard to stage or degree of severity, I prescribe the same remedies. I have long ago laid aside the traditions of my student days, which taught that salines only should be used in the acute stages, and that abortive plans were dangerous. I always use abortive measures, and mostly, I believe, succeed. At any rate, I never encounter ill consequences, and complications are rare. My prescription is a partnership of three different remedies, and it is, I believe, important that they should all be used. First, an injection of solution chloride of zinc, two grains to the ounce ; next, sandal-wood oil capsules ; and lastly, a purgative night dose, with bromide of potassium. The injection is used three or four times a day, the capsules (ten or twenty minims,) taken three times a day. The ingredients of the night dose are three drachms of Epsom salts and a half drachm of bromide of potassium. It is, I believe, the action of the last named in preventing congestion of the parts, which makes the abortive measures safe.

Moderate purgation and entire abstinence from stimulants are essential. If the case is very acute, and attended by swelling of the corpus spongiosum, I sometimes prescribe tartar emetic, or tincture of aconite, but it is very seldom, indeed, that these are necessary. If the patient be well purged, there is no risk whatever in an abortive treatment from the day he comes under treatment. The risk of orchitis, prostatitis, cystitis, etc., comes in cases which have been allowed to develop rather than in those treated abortively. I should as soon think of delaying to use local measures in gonorrhœa, as I should in purulent ophthalmia.—*Johnathan Hutchinson, in Archives of Surgery.*

